# Intracranial meningiomas: an update of the 2021 World Health Organization classifications and review of management with a focus on radiation therapy

**DOI:** 10.3389/fonc.2023.1137849

**Published:** 2023-08-22

**Authors:** Varun Yarabarla, Amrutha Mylarapu, Tatiana J. Han, Susan L. McGovern, Shaan M. Raza, Thomas H. Beckham

**Affiliations:** ^1^Philadelphia College of Osteopathic Medicine, Suwanee, GA, United States; ^2^Department of Internal Medicine, Advent Health Redmond, Rome, GA, United States; ^3^Department of Internal Medicine, WellSpan Health, York, PA, United States; ^4^Department of Radiation Oncology, The University of Texas MD Anderson Cancer Center, Houston, TX, United States; ^5^Department of Neurosurgery, The University of Texas MD Anderson Cancer Center, Houston, TX, United States

**Keywords:** intracranial meningioma, brain tumor, neurosurgery, radiosurgery, radiotherapy

## Abstract

Meningiomas account for approximately one third of all primary intracranial tumors. Arising from the cells of the arachnoid mater, these neoplasms are found along meningeal surfaces within the calvarium and spinal canal. Many are discovered incidentally, and most are idiopathic, although risk factors associated with meningioma development include age, sex, prior radiation exposure, and familial genetic diseases. The World Health Organization grading system is based on histologic criteria, and are as follows: grade 1 meningiomas, a benign subtype; grade 2 meningiomas, which are of intermediately aggressive behavior and usually manifest histologic atypia; and grade 3, which demonstrate aggressive malignant behavior. Management is heavily dependent on tumor location, grade, and symptomatology. While many imaging-defined low grade appearing meningiomas are suitable for observation with serial imaging, others require aggressive management with surgery and adjuvant radiotherapy. For patients needing intervention, surgery is the optimal definitive approach with adjuvant radiation therapy guided by extent of resection, tumor grade, and location in addition to patient specific factors such as life expectancy. For grade 1 lesions, radiation can also be used as a monotherapy in the form of stereotactic radiosurgery or standard fractionated radiation therapy depending on tumor size, anatomic location, and proximity to dose-limiting organs at risk. Optimal management is paramount because of the generally long life-expectancy of patients with meningioma and the morbidity that can arise from tumor growth and recurrence as well as therapy itself.

## 1 Introduction

Meningiomas are the most common intracranial tumors in adults, accounting for 37.6% of all primary brain and central nervous system (CNS) tumors (1). Because of the benign nature of most meningiomas and variability of treatment options, prospective and randomized clinical studies evaluating management approaches have been limited, and optimal management must be determined on a case-by-case basis. This report reviews the molecular and histologic features of meningiomas, summarizes treatment pathways for various clinical presentations including the evidence for surgical and radiotherapeutic approaches, and identifies knowledge gaps to facilitate future research in the management of meningiomas.

Meningiomas are classified according to the World Health Organization (WHO) grading system, which traditionally has been used to direct clinical prognosis and treatment. The WHO 2016 guidelines classify these tumors as one of three grades: grade 1 meningiomas are considered benign and have a low mitotic rate (<4/10 high-power field [HPF]) and no brain invasion. Grade 2 or atypical meningiomas have either a mitotic rate of 4-19/10 HPF, brain invasion, or at least 3 of 5 specific histologic features (spontaneous or geographic necrosis; prominent nucleoli; high cellularity; small cells with a high nucleus-to-cytoplasm ratio; and pattern-less sheet-like growth). Grade 3 meningiomas have either a mitotic rate >20/10 HPF or an overtly malignant appearance. Grade 3 tumors are associated with a high risk of recurrence, aggressive clinical course, and poor overall survival. The distribution of documented meningioma cases by WHO grade is 80% grade 1, 18% grade 2, and 2% grade 3 (**Table 1**) (Adapted) (1–3).

**Table 1:** Updated WHO 2021 Diagnostic Meningioma Guidelines.

	Grade 1	Grade 2	Grade 3
*Prevalence*	~85-95%	~5-10%	~1-5%
*Gender*	Female > Male	Female > Male	Female < Male
*Diagnostic Criteria*	Mitoses < 4/10 hpf	Mitoses 4 19/10 hpf OR 3/5 of the following: Necrosis High nuclear/cytoplastic ratio Prominent nucleoli Architectural sheeting Hypercellularity OR Clear cell/chordoid histology - Brain Invasion	Mitoses ≥ 20/10 hpf OR Frank anaplasia OR Papillary/rhabdoid histology
*Histologic Subtypes*	Meningothelial Fibrous (Fibroblastic) Transitional (Mixed) Psammomatous Angiomatous Microcystic Secretory Lymphoplasmacyte-rich Metaplastic	Atypical Chordoid Clear Cell	Anaplastic Papillary Rhabdoid
*Molecular Features*		4 to 19 mitotic figures in 10 consecutive HPF of each 0.16 mm^2^ (at least 2.5/mm^2^) **OR** Unequivocal brain invasion (not only perivascular spread or indentation of brain without pial breach) **OR** Specific morphological subtype (chordoid or clear cell) **OR** At least three of the following: Increased cellularity Small cells with high N:C ratio Prominent nucleoli Sheeting (uninterrupted patternless or sheet-like growth) Foci of spontaneous (non-iatrogenic) necrosis	20 or more mitotic figures in 10 consecutive HPF of each 0.16 mm^2^ (at least 12.5/mm^2^) **OR** Frank anaplasia (sarcoma-, carcinoma, or melanoma-like appearance) **OR** TERT promoter mutation **OR** Homozygous deletion of CDKN2A and/or CDKN2B
*Clinical Outcomes* *Overall Survival* *Progression-Free Survival*	80-90% 75-90%	50-79% 23-78%	14-34% 0%

### 1.1 WHO 2021 updated grading

Updated WHO guidelines were released in 2021 (**Table 1**) with noticeable changes to terminology and grading. The word ‘entity’ has been replaced with ‘type,’ and ‘variant’ has been replaced with ‘subtype’. Moreover, the use of Roman numerals in the classification system has been changed to Arabic numerals (i.e., grade II is now grade 2). The subtypes of choroid and clear cell meningiomas are assigned to at least CNS grade 2 as they have a higher likelihood of recurrence compared to grade 1 meningiomas. This classification is independent of other “atypical” features used to designate CNS WHO grade 2 for other morphologic subtypes. Another notable change involves the modifier ‘anaplastic,’ which has now been dropped in favor of grading to reflect the potential for molecular features that classify a tumor as grade 3, even in the absence of frankly anaplastic appearance on histology.

Although much of the content remains the same and histologic features remain the backbone of the classification system, the grading schema in WHO 2021 includes, for the first time, molecular factors, which can supersede histological features (4). For example, meningiomas with a telomerase reverse transcriptase (TERT) promoter mutation or a homozygous deletion of CDKN2A and/or CDKN2B are classified as WHO grade 3. The clinical data supporting inclusion of these molecular features are summarized in the next section.

### 1.2 Molecular considerations in meningioma grading and behavior

The influence of TERT promoter mutations has been observed in retrospective analyses of patient outcomes. Sham, et al. found a TERT promoter mutation rate of 6.4% among 252 patients, with enrichment in grade 2 (5.7%) and grade 3 (20%) compared with grade 1 (1.7%) (5). Notably, progression-free survival (PFS) was drastically shortened by the presence of a TERT promoter mutation, with wild-type cases progressing at a median of 179 months compared with only 10.1 months for tumors with TERT promoter mutation. Mutations were significantly associated with recurrence within individual grade groupings. This association has also been demonstrated by growing *ex vivo* patient samples *in vitro*, where TERT promoter mutations led to indefinite cell growth. Moreover, a link was noted between TERT promoter mutations and reduced PFS time and overall survival (OS) with median OS time of the entire cohort 53.8 months for patients whose tumors have TERT promotor mutation versus 115.6 for those with wild-type tumors (6).

Homozygous deletions in the cell cycle regulator genes CDKN2A and/or CDKN2B are often found in recurrent and progressive meningiomas and are associated with poor prognosis. In one study of 528 patients, 4.9% were found to have deletion of one of these genes, with significant enrichment in grade 2 (27%) and grade 3 (73%) meningiomas (7). Worse outcomes were observed for patients with the deletions, with median time to progression being 8 months versus 101 months for patients without deletions. As was the case for TERT promoter mutations, worse outcomes were observed in the entire mixed-grade cohort and within grade 2 and grade 3 cohorts when analyzed individually. Notably, CDKN2A/B deletions predicted worse outcomes in the absence of TERT promoter mutations.

While not included in the WHO 2021 guidelines as a criterion for grading, Ki-67/MIB-1, an immunohistochemical marker of cell proliferation, warrants mention as higher expression has been associated with worse prognosis. In a meta-analysis by Liu et al., 43 studies showed higher Ki-67 expression to be associated with worse OS in patients with meningiomas (8). Menger et al. studied Ki-67 labeling index for its potential to predict risk of recurrence. They concluded that the risk of recurrence after resection of meningiomas may be associated with the degree of Ki-67 positivity, with some evidence that specific values of the Ki-67 labeling index can help to predict meningioma recurrence (9).

## 2 Physiology of and risk factors for meningiomas

Meningiomas originate from the meningeal covering of the brain and spinal cord. The meninges are thought to be derived from the neural crest in the telencephalon and from the mesoderm at the skull base (10). The cells that form the layer of arachnoid mater and arachnoid villi are cytologically similar to meningioma cells and are thought to be the cells of origin of these tumors (11). Meningiomas are generally slow-growing and non-infiltrative lesions that are most often discovered incidentally during imaging, although they can present with insidious onset of symptoms related to location (12). Symptoms are generally non-specific, such as headaches due to increased intracranial pressure or generalized or partial seizures due to a focal mass effect (10). Although findings on radiographic images may indicate a meningioma, imaging cannot determine the pathologic grade of the tumor. Nevertheless, imaging surveillance is often useful in establishing the tumor’s behavior; inferring the grade of tumor is discussed in Section 3.1.

**Figure 1:**
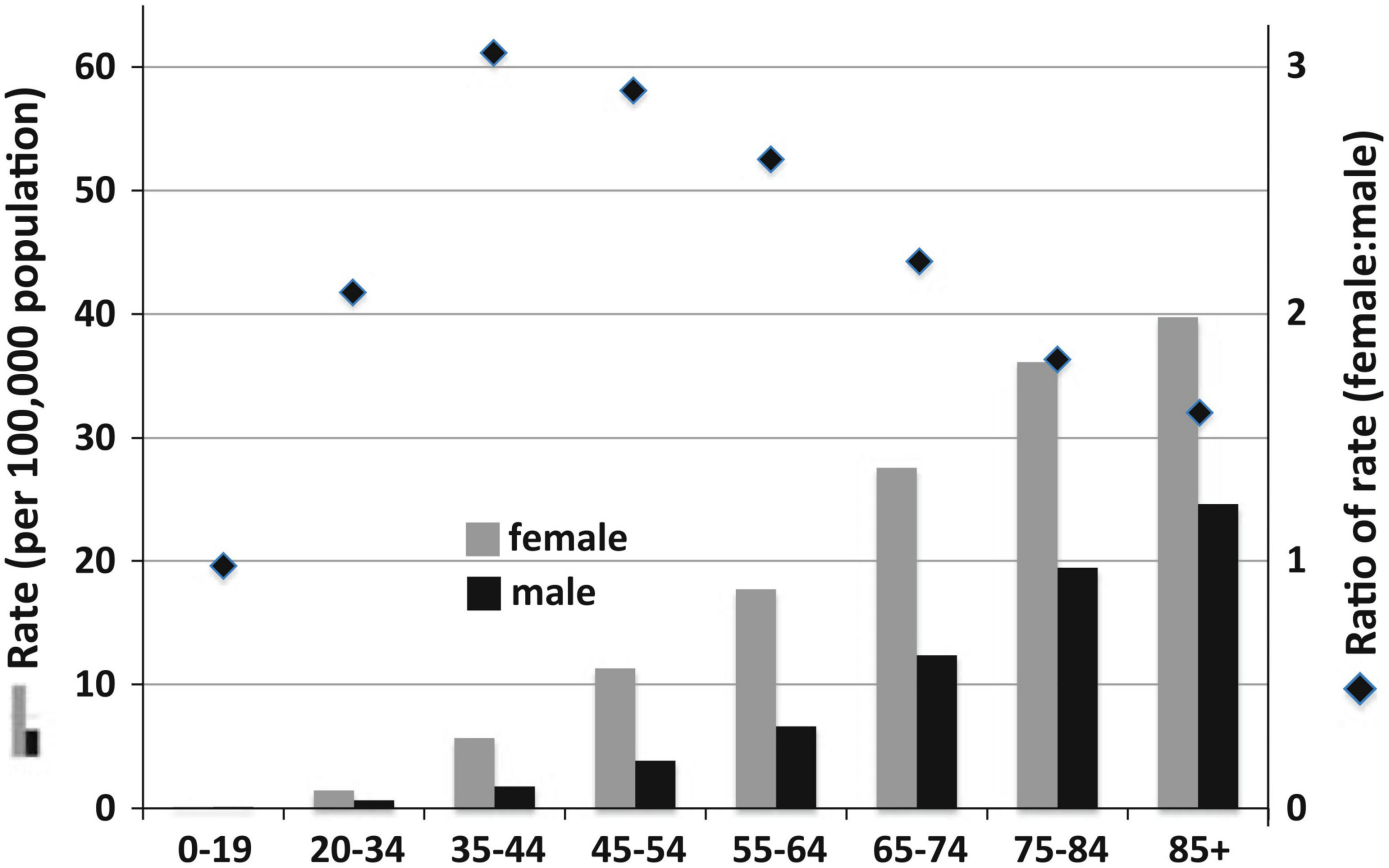
Meningioma incidence rates in the United States categorized by age and sex. (14) Diamonds and right y-axis refer to the ratio of female to male incidence per age group; the left y-axis refers to the bar graph incidence rates. The highest peak ratio of female:male incidence rate was 3.15 in the 35–44-year-old age group. (15) Reproduced and modified under the Creative Commons Attribution (CC BY) license from Wiemels J, Wrensch M, Claus EB. Epidemiology and etiology of meningioma. *J Neurooncol* 2010; 99:307–314. https://doi.org/10.1007/s11060-010-0386.

Risk factors that have been established as affecting meningioma development include age, sex, prior radiation exposure, and several genetic familial syndromes. Pediatric intracranial meningiomas are rare, accounting for only 2.5% of total cases; meningiomas most often occur in individuals aged 40 to 70 years (13). With a pathological prevalence of 1/100 in the United States, females are twice as likely as males to develop meningioma, and are 3.15 times more likely to develop meningiomas during their peak reproductive years because of cyclic hormonal exposure. Meningiomas can express estrogen, progesterone, and androgen receptors; indeed, the involvement of endogenous and exogenous hormones is thought to contribute to their increased incidence in females (**Figure 1**) (15).

Prior radiation exposure, particularly in childhood, is another known risk factor for intracranial meningiomas. In one Israeli study between 1948 and 1960 of radiotherapy exposure in a large population of children treated with low-dose radiation for tinea capitis, the relative risk of meningioma development later in life was found to be 9.5 compared with age-matched controls (16). Other population studies of radiation exposure through diagnostic imaging (i.e., dental x-rays) reveal a higher risk in patients exposed to radiation at young ages (17).

Several studies have examined the role of certain gene mutations in the development of radiation-induced meningiomas in comparison with sporadic meningiomas. Radiation-induced meningiomas are typically more aggressive, with anaplastic or atypical histologic characteristics, and tend to have higher rates of recurrence (18, 19). A significant interaction was observed between radiation exposure and single-nucleotide polymorphisms in cyclin D1 and p16 (*P*=0.005 and *P*=0057), indicating their potential involvement in modifying the risk of meningiomas in irradiated vs non-irradiated individuals. Both of these genes are involved in the regulation of the cell cycle control pathway, specifically the transition from G1 to S phase (20, 21). In other studies, chromosomal losses of 1p and 22q were more commonly observed in radiation-induced meningioma compared with the spontaneous type, (21) and Shoshan et al. and Jochaim et al. both noted that NF2 gene inactivation was less likely to be detected in radiation-induced meningiomas (22, 23). These observations indicate distinct differences in the histologic and clinical characteristics of radiation-induced meningioma compared with spontaneous meningioma.

Several genetic conditions have been associated with development of meningiomas, the best-known being mutations in neurofibromatosis genes. Although neurofibromatosis type 2 (NF2) is much less common than neurofibromatosis type 1 (NF1), NF2 is more commonly associated with meningiomas, with estimates that 45%–58% of NF2 patients harbor intracranial meningiomas and 20% harbor spinal meningiomas (24). This association and progression of meningiomas in NF2 patients is linked with the NF2 gene mutation, as studies have shown that NF2 serves as a tumor suppressor in meningioma tumorigenesis (25). Beyond neurofibromatosis, many other familial syndromes are linked with evidence of increased chances of developing an intracranial meningioma, including nevoid basal cell carcinoma syndrome, multiple endocrine neoplasia 1 (MEN1), Cowden syndrome, Werner syndrome, BAP1 tumor predisposition syndrome, and Rubinstein-Taybi syndrome (26, 27). Despite associations between these syndromes and predisposition to meningiomas, most meningiomas occur sporadically without a readily identifiable environmental exposure or familial component, indicating that meningioma development is highly multifactorial (12).

## 3 Management options

### 3.1 Observation

The appropriate management strategy for meningiomas must be determined on a case-by-case basis. For patients with incidentally discovered grade 1 tumors in low-risk locations, observation with serial magnetic resonance imaging (MRI) is often the recommended initial approach for both diagnosis and management. Asymptomatic lesions that have characteristics of a meningioma on MRI are often found to have a slow growth rate consistent with a benign meningioma on serial imaging. Currently, MRI is the gold standard imaging technique for characterizing meningiomas. Meningiomas present as hypo- to iso-intense on T1-weighted sequences, with variable signal on T2-weighted sequences and avid homogenous uptake of contrast (28).

Clinically, it is important to consider other etiologies (such as metastasis) that could appear as a dural-based lesion that can mimic meningioma radiologically. Biopsy or resection provides a definitive histologic diagnosis; however, in many cases lesions are diagnosed based on imaging characteristics alone. In some cases, advanced imaging such as DOTATATE positron emission tomography (PET) scans can be helpful for confirming the diagnosis of a meningioma, which often express somatostatin receptors and are typically DOTATATE-avid. Use of somatostatin receptor II ligands radiolabeled with gallium-68 has shown higher sensitivity for detecting meningiomas relative to contrast-enhanced MRI. One retrospective analysis showed an overall detection rate of 92% using PET/computed tomography (CT), as compared with an overall detection rate of 90% for contrast-enhanced MRI (29). The use of molecular imaging can particularly useful for evaluating the extent of disease in anatomically complex locations such as the base of skull. For cases in which distinguishing tumor from surgical changes is difficult, DOTATATE-PET can provide further diagnostic information and has been demonstrated to be useful in radiotherapy planning. In addition to imaging guided radiation treatment, DOTATATE-PET imaging may be useful for evaluation of the extent of surgical resection (30, 31).

**Figure 2:**
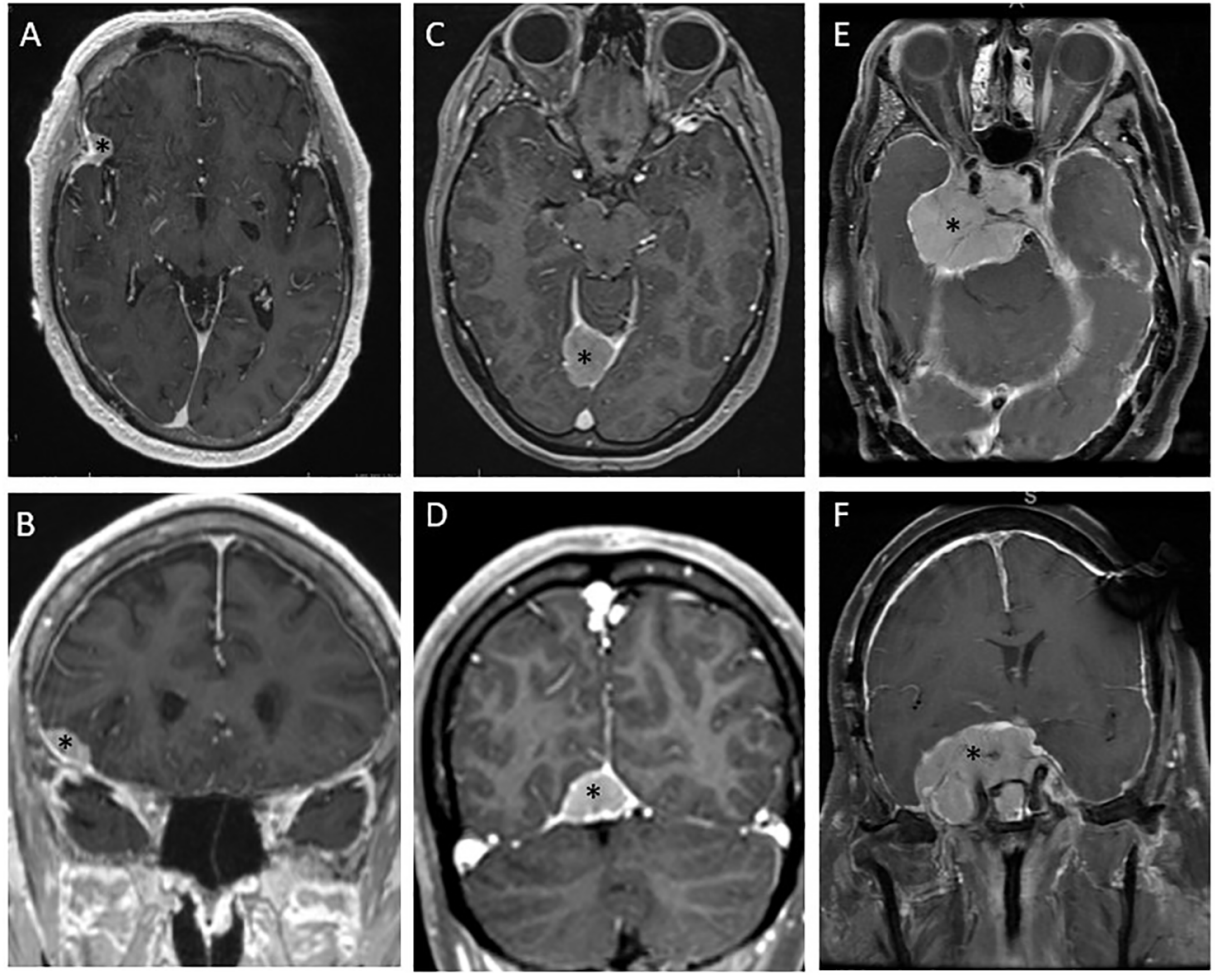
Diverse presentations of and management strategies for meningiomas. **(A, B)** Axial and coronal magnetic resonance (MR) images depict a presumed grade 1 meningioma incidentally discovered on a fluciclovine positron emission tomography (PET) scan for metastatic prostate cancer. The low-risk location was thought to make this lesion amenable to observation, and the lack of growth on the 2-month MR images increased confidence in the diagnosis of low-grade meningioma as opposed to dural-based metastasis. This lesion was considered appropriate for observation. **(C, D)** Axial and coronal MR images depict a tentorial meningioma that had previously been subtotally resected. This lesion continued growing after resection and was then treated with single-fraction radiosurgery. **(E, F)** Axial and coronal MR images depict a large suprasellar base-of-skull meningioma involving the right cavernous sinus and encasing the optic chiasm with a mass effect on the brainstem. This lesion had been subtotally resected 5 years previously and grew very slowly, eventually causing visual field cut. This lesion was treated with conventionally fractionated radiotherapy. *denotes the location of the tumor.

The choice of treatment for a grade 1 meningioma with demonstrated indolent behavior based on serial MRI should consider the patient’s age, life expectancy based on comorbid conditions, and probability of morbid progression based on tumor location. Most benign meningiomas have a volumetric growth rate of 5.82% per year, (32) so a small lesion in a non-critical anatomic location can be expected to have a low risk of morbid progression, and observation should be the first step in management. **Figures 2A, B** demonstrate a patient with metastatic prostate cancer in whom a fluciclovine PET revealed an avid lesion in the right pterion. MRI showed a dural-based lesion consistent with meningioma; however, in a patient with metastatic cancer, it is important to consider the possibility of metastasis. In this patient, the low-risk location and small size of the lesion made interval MRI a reasonable observation strategy despite the possibility of a dural based metastasis, and subsequent indolent behavior on MRI was consistent with a low grade meningioma. A DOTATATE PET scan would also have been a reasonable step to increase confidence in the diagnosis of meningioma. A small lesion with a slow rate of growth on serial imaging does not need treatment and can be observed.

In contrast, lesions in anatomically complex sites such as the skull base that have demonstrated growth may be better served with local therapy despite the lack of symptoms, or very early in the development of symptoms. Objective size- or growth-based recommendations are difficult to provide because of patient- and tumor-specific nuances, but typically observation is not recommended as a long-term option for larger tumors or those with a more rapid rate of growth (33). Observation is not a reasonable strategy for patients with grade 1 tumors with symptoms related to the mass, for grade 1 tumors in locations at risk for symptomatic local progression that can lead to morbidity over the course of the patient’s predicted lifespan, and grade 2 or 3 lesions because of their locally aggressive behavior and malignant potential.

### 3.2 Extent of surgical resection and impact on risk of recurrence

Surgical resection is the preferred first step in management for lesions unfit for observation, including suspected grade 2 or 3 lesions. Surgery provides immediate and durable decompression of mass effect and permits histologic diagnosis. The extent of surgery has classically been graded according to the Simpson criteria, which is based on an intraoperative visual assessment of surgical resection, ranging from grade I to grade V. Simpson grade I indicates “macroscopically complete removal of tumor, with excision of its dural attachment, and of any abnormal bone. Includes resection of venous sinus if involved,” whereas Simpson grade V indicates “simple decompression, with or without biopsy” (34). Grades II and III indicate gross removal of all tumor with coagulation or resection of the dural attachment (grade II) or simply gross total resection (GTR) (grade III). In the literature, grade I-III tumors are typically considered “GTRs.” Simpson grade has also correlated with risk of symptomatic recurrence: at 10 years, the risk of recurrence was 9% for grade I tumors, and 19% for grade II tumors. Because grades III-V tumors were not appropriate for radical surgery, symptomatic recurrence was considered highly likely (34).

The Simpson grading criteria is still in use as a predictor of recurrence, although a modern view must consider more features to accurately assess risk of recurrence postoperatively. Simpson’s original report from 1957 predates WHO grading by decades, so it does not consider pathologic grading or molecular features, which are strongly associated with risk of recurrence. Dr. Simpson does describe the histologic features of many cases in his report and makes several prescient observations associated with risk of recurrence now present in WHO grading such as mitotic features, invasiveness, histologic subtypes, and malignant appearance. These patients were also treated in an era without MRI or computed tomography (CT), which is useful in determining the presence and degree of residual tumor. A number of reports have sought to evaluate the validity of Simpson grading in the modern era with attention to WHO grade. A report of 113 WHO grade 1 tumors between 1991-2000 who received no adjuvant radiation therapy found Simpson grade still correlated well with risk of recurrence (35). An experience from the Barrow Neurologic Institute of WHO grade 1 tumors between 2007 and 2017 also found validity to the Simpson grade with each grade I-IV significantly associated with a higher risk of recurrence. Grade IV patients were frequently treated with SRS, with irradiated grade IV patients having similar risk of recurrence to Grade II and III highlighting the role of adjuvant radiotherapy in reducing the risk of recurrence after less aggressive surgeries in the modern era (36).

A useful set of guidelines comes from the European Association of Neuro-Oncology who suggest follow-up intervals for resection based on the Simpson grading criteria. Specifically, WHO grade 1 tumors that can be completely resected (Simpson grade I-III) can be observed at 3-month follow-up intervals for 5 years. Because the Simpson grade refers to the extent of resection, which can affect the rate of recurrence, the risk of recurrence becomes a qualifying factor in determining the use of adjuvant therapy (34).

While there may be controversy about the goal of surgery and the validity of the Simpson grading in the modern era, when taken as a single risk factor it seems the degree of surgical resection still holds value in the estimation of post operative risk of recurrence. Whether the goal of surgery in all cases should be Simpson grade 1 now that imaging surveillance is superior and effective adjuvant therapies may reduce the need for aggressive surgery is a complex question and the source of much debate which is outside the scope of this review (37–40).

### 3.3 Adjuvant radiation therapy

Several factors contribute to the decision of whether to use adjuvant radiotherapy after surgical resection. Given the limited prospective data regarding management strategies and wide variation in approaches because of differences in patient factors (e.g., age, comorbidities) and disease-specific features (e.g., size, grade, and location) that influence the feasible extent of resection, no universally accepted guidelines have been established. The need for adjuvant therapy is judged based on the risk and potential consequences of local progression after surgery. Tumor grade is a useful starting point for discussing the need for adjuvant radiotherapy.

#### 3.3.1 WHO grade 1

As discussed in previous sections, grade 1 meningiomas are benign and the need for resection is typically dictated by symptoms, either current or anticipated in the event of tumor progression. After surgery that achieves the goal of therapy, whether that is decompressing the brain parenchyma or reducing the influence of gross disease on the brainstem or cranial nerves, often the most reasonable approach is to resume observation, even in the case of subtotal resection (STR) (41). Exceptions to this include cases in which symptoms remain due to residual disease, or situations in which the probability of symptom development with disease growth is high and re-resection is unlikely to be feasible. This can be the case with skull-based meningiomas, for which GTR is difficult and symptoms often reflect involvement of several cranial nerves and neural foramina. Patient-specific factors can also be considered. For example, in patients who are elderly or whose life expectancy is limited because of comorbidities, slow progression of meningioma may be less likely to result in symptoms during the remaining lifespan of the patient. This should be considered in the decision to proceed with surgery and can also factor into the decision for adjuvant radiation.

#### 3.3.2 WHO grade 2

These meningiomas have a higher risk of recurrence after both GTR and STR, which must be considered when considering adjuvant radiotherapy. The reported risk of recurrence after GTR for an atypical meningioma without adjuvant therapy is significant at more than 40% by 5 years (42, 43). Thus even after a GTR, adjuvant radiation is often used to improve local recurrence rates. In one such study, Komotar et al. found that after GTR, the local recurrence rate at 44 months was 41% for patients who did not undergo adjuvant radiotherapy as opposed to 8% among those who did (43). In a single-institution report of 262 patients, Chen et al. found that adjuvant radiotherapy improved local control in a mixed group of patients with atypical meningioma regardless of extent of resection (STR or GTR) when controlling for several relevant clinical and pathologic factors (44).

In contrast, other series have found no objective benefit from radiotherapy after GTR of grade 2 meningiomas. In one report of 71 patients who had GTR for atypical meningioma, the local recurrence rate at 5 years was 16.7% for patients who did not receive adjuvant radiotherapy as compared with 11.8% in 31 patients who did (45). These investigators further identified 5 factors predictive of tumor recurrence—mitotic index, sheeting, necrosis, non-use of adjuvant radiotherapy, and STR. Other groups have attempted to use pathologic features to risk-stratify patients after GTR for atypical meningiomas. Fioravanzo et al. led such an effort and created a clinicopathological risk score system in which one point was assigned for each of 5 high-risk parameters: male sex, parasagittal site, Simpson grade III resection, mitotic index ≥6/10 HPF, and sheeting (46). A score of ≥2 was associated with a nearly 5-fold risk of shorter disease-free survival and was suggested as a way to help risk-stratify patients in need of adjuvant radiotherapy.

A meta-analysis of relevant studies found significantly lower local recurrence rates (18% vs 31%) when using WHO grade 2 criteria from 2007 or 2016 (which include brain invasion as a factor for diagnosing grade 2) versus earlier grading systems that did not consider this factor. They also found that receipt of adjuvant radiotherapy led to improved PFS relative to observation only (47). However, another recent meta-analysis of 3500 patients from 25 studies found no significant association between brain invasion and mitoses, finding that only spontaneous necrosis was associated with PFS (48).

A far less controversial approach is to routinely offer adjuvant radiation after STR for WHO grade 2 meningiomas. Local recurrence rates are very high, and adjuvant radiotherapy is effective in reducing them. In one such study, use of adjuvant radiotherapy after STR reduced the risk of recurrence from 91% to 20% (45). A National Cancer Data Base analysis by Wang et al. found that adjuvant radiotherapy conferred an OS benefit for patients with WHO grade 2 meningioma after STR (49). A survival benefit was not seen for patients after GTR, but the effects of adjuvant radiotherapy on other measures such as local recurrence and PFS were not analyzed.

While the benefit of adjuvant radiotherapy for grade 2 meningiomas after GTR can be supported or refuted by numerous institutional series, we eagerly await the results of NRG BN003, a phase III randomized trial of adjuvant radiotherapy versus observation after GTR for grade 2 meningiomas (NCT03180268).

#### 3.3.3 WHO grade 3

Grade 3 meningiomas are malignant lesions that demonstrate aggressive local invasion of the brain, bone, and soft tissue and exhibit metastatic potential (50, 51). Given the high recurrence rates after surgery alone, nearly all cases of grade 3 meningioma should receive postoperative radiotherapy to reduce the risk of recurrence. Nevertheless, outcomes remain poor even with maximum surgery and postoperative radiotherapy.

At many institutions, findings from RTOG 0539 have been used to codify the approach to adjuvant therapy and have been essential for developing prospective trials of adjuvant radiotherapy for meningiomas. RTOG 0539 was a phase II prospective study that described three risk groups and therapeutic approaches for meningiomas after surgery:

***Low-risk***. Grade 1 tumors, whether GTR or STR, were observed after surgery with no adjuvant radiotherapy. Of the 92% of patients with a GTR, the 3-year PFS rate was 91.8% and the 5-year PFS rate was 86.1%. When this review was written, these outcomes had been presented only in abstract form (52).
***Intermediate-risk***. Patients with newly diagnosed grade 2 tumors after GTR, or recurrent grade 1 tumors after either type of resection, were treated with adjuvant radiotherapy, 54 Gy in 30 fractions to the tumor bed and residual disease plus a 1-cm clinical target volume (CTV) along at-risk surfaces. The 3-year PFS rate was excellent at 93.8%, which exceeded the 3-year findings from the low-risk group (53).
***High-risk***. New or recurrent grade 3 tumors regardless of resection extent, recurrent grade 2 tumors, or STR grade 2 tumors were all considered high risk. Patients with high-risk tumors were treated with 60 Gy in 30 fractions with a simultaneous integrated boost technique, in which 60 Gy was delivered to the gross disease and cavity plus a 1-cm CTV and 54 Gy was delivered to a 2-cm CTV with respect to barriers of spread. At 3 years, the PFS rate was 59.2% and the OS rate was 78.6%, highlighting the significantly worse outcomes for patients fitting the high-risk category (54).

## 4 Radiation monotherapy in meningiomas

The optimal management for grade 1 meningiomas is complete resection of the tumor, but not all tumors are amenable to surgical resection, and not all patients are suitable for surgery. Moreover, even benign meningiomas have a long-term risk of local recurrence without adjuvant radiotherapy. For low-grade tumors that are not likely to be completely resected, or for poor surgical candidates, radiation monotherapy, whether as stereotactic radiosurgery or fractionated stereotactic radiotherapy, is an excellent alternative (55).

### 4.1 Stereotactic radiosurgery

Stereotactic radiotherapy (SRS), delivered in as few as 1 and as many as 5 fractions, is a form of highly conformal, high-dose, image-guided radiotherapy. Single-fraction SRS is often an option for smaller tumors (typically <2 cm in diameter) and tumors at acceptable distance from critical organs at risk such as the optic chiasm and brainstem. Fractionated stereotactic radiotherapy, often given as 3 or 5 treatments, is sometimes used for larger lesions. SRS can be done *via* linear accelerator-based SRS or Gamma Knife radiosurgery. Generally, SRS is used when surgical risk is high because of the location of the meningioma, because the patient’s suitability for surgery is not optimal, or when a distinct tumor border is present with a maximum diameter of <3-4 cm (56). Reported series have shown SRS to provide excellent local control rates. In a seminal report of 251 patients by Pollock et al., SRS for patients with imaging-defined meningiomas without surgical resection led to a local control rate of 99.4% at 10 years (57). In an earlier series, the same group found SRS to provide equivalent outcomes as those of a Simpson I resection for benign meningiomas <3.5 cm, and superior to less extensive resections (58). A study reported by Bir et al. concluded that Gamma Knife surgery led to higher rates of locally controlled tumor growth at 5 years and at 10 years compared with surgical resection. **Figures 2C, D** illustrate an example of a lesion managed with SRS. This lesion was first partially resected and confirmed to be a grade 1 meningioma. After a period of observation, the lesion was found to grow to an extent that, given the patient’s relatively young age, posed a clinically significant risk over their lifetime. That lesion was treated with SRS, 12 Gy to the 50% isodose line, by using Gamma Knife.

Data for SRS for treatment of grade 2 and 3 meningiomas is less robust and clinical results are poorer. Treatment of grade 2 or 3 meningiomas with SRS has led to local control rates of 50%-70% (59–61). Zhang et al. reported a 5-year local control rate of 48% for radiation-naïve patients and 0% for patients with prior radiation exposure (60). Another single-institution series of 183 lesions in 48 patients demonstrated local and marginal control rates of 42% at 5 years after SRS (61). A meta-analysis of 19 studies including 647 patients with grade 2 or 3 meningiomas found a wide range of outcomes, which reflects the selection bias inherent in single-institution studies (62). For grade 2 tumors, 5-year PFS rates ranged from 25% to 83%, but for grade 3 the range was from 0% to 72%. Thus, although the available data do not support the routine use of radiosurgery for grade 2 or 3 meningiomas, well-selected recurrent cases may benefit from SRS in that some patients can achieve meaningful PFS.

Fractionated stereotactic radiotherapy may be an alternative for meningiomas when single fraction SRS is not ideal because of lesion size or proximity to organs at risk. Marchetti et al. reported on 143 patients with sellar and parasellar meningiomas presumed to be grade 1 upon imaging that were treated with 25 Gy in 5 fractions; this approach led to an impressive 5-year PFS rate of 93% (63). Interestingly, among patients with tumors close to the optic pathway, 36% had improved visual function as compared with 7.4% having worsened visual function, highlighting the favorable safety profile of fractionated stereotactic radiotherapy. A propensity-score matched analysis of 145 cases treated with SRS (12-16 Gy) and 81 treated with fractionated stereotactic radiotherapy (21 Gy in 3 fractions) found no statistically significant differences in local control at 2 years, 5 years, or 10 years (64). Numerically, 10-year local control rates favored fractionated stereotactic radiotherapy at 91% versus 74% with SRS. A meta-analysis of hypofractionated radiation for meningiomas reported local control rates of 90%-100% from 10 studies for which data were available (65).

Identifying patients who are suitable candidates for SRS is crucial, as the high dose given per fraction presents a set of unique challenges. Among patients who underwent SRS in one study, approximately 9% had treatment-related complications such as radiation necrosis, cranial nerve deficit, motor deficit, hydrocephalus, vascular occlusion, and development of peritumoral edema (66). Despite these risks, experienced radiosurgeons can effectively identify patients for whom SRS is suitable, with an acceptable risk-benefit ratio. Moreover, radionecrosis and edema are commonly asymptomatic and self-limited, and symptoms can be effectively managed for the vast majority of patients.

### 4.2 Conventionally fractionated radiotherapy

Although SRS and fractionated stereotactic radiotherapy are convenient and effective approaches for many meningiomas, they are not suitable in all situations. For some lesions, size alone may limit the ability to use SRS without an unacceptably high risk of radiation necrosis. Also, many lesions are intimately associated with at-risk structures such as the brainstem, skull base neural foramina, cranial nerves, cochlea, and orbits. For such cases, conventionally fractionated radiotherapy is preferable. Conventional fractionation regimens vary widely between institutions and are modified for specific clinical scenarios. A typical conventional fractionation schedule refers to delivery of 1.8- to 2.0-Gy fractions to total doses ranging from 50.4 Gy to 60 Gy. **Figures 2E, F** illustrate a lesion that required fractionated radiotherapy owing to extensive involvement of the skull base, encasing radiation-sensitive critical anatomy including the optic chiasm and having a broad mass effect on the brainstem.

Like the experience with SRS for presumed benign lesions, local control after radiation alone for imaging-defined meningiomas has also been quite high. In one report of 57 patients with imaging-defined lesions of the cavernous sinus, Milker-Zabel et al. reported a local control rate of 100% at a median follow-up time of 6.5 years. The high-risk location of the lesions meant that most patients had pre-existing neurologic symptoms, and in 11 of those patients, neurologic symptoms improved after radiotherapy (67). Morgan et al. compared conventionally fractionated radiotherapy with SRS for imaging-defined meningiomas and found similar rates of local control (97.8% and 94.6% at 5 years) between the two modalities. However, conventionally fractionated radiotherapy was rarely associated with treatment-related edema (2.5% at 2 years) compared with SRS (34.6% at 2 years). Although this finding reveals little about the quality of life for patients receiving either modality, it is generally accepted that fractionated therapy is less likely to result in damage to organs at risk such as optic structures, brainstem, and the brain itself compared with SRS, so the surrogate of late therapy-related edema is interesting. Importantly, lesions selected for conventional fractionated radiation are usually much larger; in the study reported by Morgan and colleagues, lesions to be treated with fractionated stereotactic radiotherapy were 15 mL versus 5 mL for lesions to be treated with SRS (68).

Conventionally fractionated radiotherapy given with definitive intent (as opposed to adjuvant therapy, as discussed in Section 3.3), uses strategies to target lesions by tumor grade similar to those described in RTOG 0539, that is, larger margins for subclinical tumor spread are typically used for grade 2 and 3 tumors. Moreover, although grade 1 tumors do not invade the brain parenchyma, grade 2 and 3 tumors can, and so the CTV is extended into the brain parenchyma as well as along the adjacent dura. Overall, this typically results in larger target volumes for grade 2 and 3 lesions.

While local control for grade 1 tumors is excellent with a variety of radiation approaches, outcomes including local control for grade 2 and 3 tumors have been less impressive, as demonstrated by the high-risk arm of RTOG 0539. This finding has led to efforts to escalate the total radiation dose. In one study of 31 patients with grade 2 or 3 meningiomas, total doses of >60 Gy were associated with improved local control and survival (69). In a similar study of patients treated with combined proton/photon external-beam radiation, Boskos et al. also found improved OS when radiation doses exceeded 60 Gy (70). Dose escalation beyond 60 Gy for grade 2 and 3 tumors is being investigated in a phase I/II study of intensity-modulated proton therapy (NCT02693990).

## 5 Recurrence after initial local therapy

Treatment for recurrent meningiomas will vary depending on whether radiation was previously used as a treatment option. If no prior radiation was given, then the usual options to be considered would be repeat surgery or radiation. For recurrent grade 1 meningiomas, considerations for use of local therapy for recurrent disease are similar to those for determining the need for local control: Is the tumor causing symptoms? Is it likely to cause symptoms if it continues to progress?

Recurrent grade 2 and 3 meningiomas are more challenging and may behave more aggressively with increased rates of locoregional and distant intracranial failure. Chen et al. described 65 patients with atypical meningiomas undergoing salvage therapy with surgery, radiation, or both (71). Interestingly 39% of patients had three or more recurrences, with multifocal local recurrence the most strongly associated with further recurrence after salvage therapy. Intervals to subsequent recurrence also tended to shorten over time, highlighting an accelerating clinical course with reduced disease-free survival time between recurrences. Freedom from local failure rates at 2 years have ranged from 36% to 73% depending on the modality used.

Salvage strategies involving re-irradiation depend principally on whether meaningful additional doses of radiation could be delivered with an acceptable risk profile. In challenging clinical situations, careful consideration should be given to patient-specific factors, recurrence location, symptoms, and risk from additional radiation.

Specialized radiation modalities may offer an improved therapeutic ratio for reirradiation. Particle therapy with proton or carbon ions has the advantage of lower integral doses to non-target tissues and is often considered in cases of reirradiation for this reason (72). El Shafie et al. described 42 patients treated with carbon or proton therapy for re-irradiation. Although their patients had diverse tumor locations and grades, the reported PFS rate of 71% at 1 year was remarkable for a group in which most patients had grade 2 or 3 meningiomas.

Brachytherapy can also be considered for reirradiation among patients undergoing additional resection. Brachytherapy offers an attractive combination of rapid dose falloff from the positioning of the radiation source near the target tissue and the convenience of completing radiation during the surgery, eliminating the need to return for fractionated postoperative radiotherapy. In one series of 15 patients with recurrent grade 2 or 3 meningioma in whom Cs-131 or I-125 stranded seeds were placed in the operative cavity at resection, local control was poor, with all but 2 patients experiencing local failure, and repeat surgery for wound infection was required for 6 patients (40%) (73). Recently, GammaTile (collagen tile Cs-131 brachytherapy) has been approved for use by the US Food and Drug Administration for recurrent brain tumors, largely on the basis of safety and efficacy findings in previously irradiated meningiomas (74). In that study, the local control rate was 90% at a median follow-up time of 15 months, which shows impressive control of lesion growth considering that 80% of patients had grade 2 or 3 lesions at the time of brachytherapy. Two of those 19 patients experienced radiation necrosis that was treated medically, and another 2 had surgery for complications; however, given the heavily pretreated nature of the population, the safety profile and efficacy seem promising. Additional studies are needed to determine whether these promising early results and favorable safety profiles hold up over time as more patients are tested.

## 6 Systemic therapy in meningiomas

Systemic therapy plays a limited role in meningioma management and is typically used in the salvage setting when local therapy is not feasible. Evidence for the effectiveness of systemic therapy approaches is extremely limited and most clinical studies have been retrospective or observational. The National Comprehensive Cancer Network (NCCN) guidelines recommend chemotherapy for disease progression in cases when additional resection or radiotherapy is not possible. NCCN guidelines based on lower-level evidence recommend the use of vascular endothelial growth factor (VEGF) inhibitors (e.g., sunitinib), bevacizumab in combination with everolimus, or, in certain circumstances, somatostatin receptor agonists (e.g., octreotide). Patients with radiographic progression treated with bevacizumab, alone or in combination with everolimus, have shown slowing of neurologic decline (75–81). Unfortunately, to date several systemic drugs, including dacarbazine, doxorubicin, cyclophosphamide, vincristine, and temozolomide, have shown virtually no benefit, (82, 83) and smaller studies of anti-angiogenic drugs have not produced confirmatory findings. One small study of 8 participants evaluated the use of bevacizumab and everolimus and found no objective tumor response, but disease stability was prolonged (79).

Other recent studies have examined the value of traditional cytotoxic chemotherapy agents in the treatment of meningiomas. For example, trabectedin, an anticancer compound isolated from the sea squirt *Ecteinascidia turbinate*, was found to enhance the cytotoxic activity of hydroxyurea, doxorubicin, and cisplatin in patients with higher-grade meningiomas (84). In another study, an *in vitro* review of animal and human studies revealed the potential of chemotherapy as a second or third-line treatment for benign meningiomas. That study demonstrated the importance of considering histopathologically benign variants, as this characteristic may be significant in tumor responsiveness to specific chemotherapeutic agents (85).

## 7 Future outlook

Efforts to conduct large clinical trials of meningioma management strategies have been hindered by the complex and variable nature of this disease. The ideal treatment course should be based on the available data, both retrospective and prospective, but should also be modified on a case-by-case basis with consideration of patient- and tumor-specific factors. The complex nature of meningiomas tends to require a personalized and multidisciplinary approach to optimal management in which regardless of treatment choice, the primary goal is to preserve patients’ quality of life and maintain neurological function while minimizing the risk of adverse effects of treatment (86). Currently accruing BN003, which randomizes patients with grade 2 meningiomas after GTR to observation versus adjuvant radiation is a welcome effort to provide high quality data for a very controversial clinical presentation. While radiotherapy is quite effective in treating meningiomas, as with most adjuvant therapies, many patients who receive it would not have recurred and thus derive no benefit from therapy. They are, however, at risk for toxicity from treatment. Well-designed randomized trials are essential to help clinicians and patients understand the magnitude of benefit and risk associated with a therapy choice.

As our understanding of the molecular underpinnings of meningiomas grows deeper, as reflected in the WHO 2021 updated classifications, there is reason for optimism that features beyond WHO grade and extent of surgical resection will continue to improve our ability to risk stratify patients and guide adjuvant therapy. Prospective and randomized studies are a valuable opportunity to correlate molecular features with clinical outcomes to help develop the next generation of questions to address.

For meningiomas that have recurred or progressed after surgical resection and radiosurgery, the next step may involve systemic therapy to modulate tumor growth. Although systemic therapeutic options at this time are limited in both efficacy and evidence, molecularly targeted therapies may offer hope for some patients. A trial sponsored by the Alliance for Clinical Trials in Oncology Group and the National Cancer Institute is looking into potential molecular therapeutic targets, and is recruiting patients with mutations in AKT1, SMO and NF2 for treatment with AKT, SMO, and FAK inhibitors, respectively (10). Outcomes data for these patients are still being collected, and no preliminary analyses have been reported (87). In the era of molecularly targeted cancer therapy, patient-specific mutation analysis may guide the choice for off label use of targeted therapies. While evidence is currently limited, there are numerous clinical trials underway investigating targeted therapies, immunotherapies, and theragnostic approaches, summarized in **Table 2**. Given the lack of treatment options for meningiomas once local therapies have been exhausted, case reports and series of success and failure with systemic therapy approaches will be welcome as we await reports from clinical trials.

**Table 2:** Actively recruiting clinical trials for meningioma treatment.

Trial ID	Study Title	Population and Study Design	Intervention	Primary Endpoint(s)
NCT02648997	An Open-Label Phase II Study of Nivolumab in Adult Participants With Recurrent High-Grade Meningioma	Phase 2: Recurrent High-Grade Meningioma	Drug: Nivolumab - 240 mg Drug: Ipilimumab - 1 mg/kg Drug: Nivolumab - 480 mg Drug: Nivolumab - 3 mg/kg Radiation: External Beam RT	PFS
NCT04997317	Treatment of Recurrent or Progressive Meningiomas With the Radiolabeled Somatostatin Antagonist 177Lu-satoreotide	Phase 1: Recurrent or Progressive Meningioma	Drug: 177Lu-DOTA-JR11 (Phase 0); Cycle 1 and Cycle 2 (cross-over) Drug: 177Lu-DOTATOC (Phase 0); Cycle 1 and Cycle 2 (cross-over), Cycle 3 and 4 Drug: 177Lu-DOTA-JR11 (Phase I/II)	Therapeutic Index, PFS
NCT03971461	A Single Arm, Open-label, Multicenter Phase II Study of 177Lu-DOTATATE Radionuclide in Adults With Progressive or High-risk Meningioma	Phase 2: Progressive or High-Grade Meningioma	Lutathera	PFS, ORR, OS
NCT03631953	Combination of Alpelisib and Trametinib in Progressive Refractory Meningiomas (ALTREM)	Phase 1: Progressive Meningioma	Drug: Trametinib Trametinib administered at a fixed dose (1.5 mg daily) Drug: Alpelisib A panel of 3 doses of ALPELISIB could be tested	DLT
NCT02523014	Vismodegib, FAK Inhibitor GSK2256098, Capivasertib, and Abemaciclib in Treating Patients With Progressive Meningiomas	Phase 2: Progressive Meningioma	Drug: Vismodegib Drug: FAK Inhibitor GSK2256098 Drug: Capivasertib Drug: Abemaciclib	PFS, ORR, OS
NCT03604978	Nivolumab and Multi-fraction Stereotactic Radiosurgery With or Without Ipilimumab in Treating Patients With Recurrent Grade II-III Meningioma	Phase 1/2: Grade 2, Grade 3, and Recurrent Meningioma	Drug: Ipilimumab Drug: Nivolumab Radiation: Stereotactic Radiosurgery	Therapeutic Index, PFS, ORR, OS
NCT04082520	Lutathera for the Treatment of Inoperable, Progressive Meningioma After External Beam Radiation Therapy	Phase 2: Grade 1-3, Recurrent, Unresectable Meningioma	Drug: Lutetium Lu 177 Dotatate Radiation: Gallium Ga 68-DOTATATE	PFS, OS
NCT04659811	Stereotactic Radiosurgery and Immunotherapy (Pembrolizumab) for the Treatment of Recurrent Meningioma	Phase 2: Grade 1-3, Recurrent Meningioma	Drug: Pembrolizumab Procedure: Stereotactic Radiosurgery	PFS, OS
NCT04728568	Exploratory Study of PD-1 Neoadjuvant Treatment of Recurrent Meningioma	High Grade Meningioma	Drug: Sintilimab	PFS, OS
NCT04501705	Apatinib in the Treatment of Recurrent Atypical/Malignant Meningioma in Adults	Recurrent, High-Grade Meningioma	Drug: Apatinib Mesylate	PFS, ORR, OS

PFS, Progression-Free Survival; ORR, Objective Response Rate; OS, Overall Survival; DLT, Dose Limiting Toxicity.

PFS, progression-free survival; ORR, objective response rate; OS, overall survival; DLT, dose-limiting toxicity.

Given that most meningiomas typically affect outcomes and quality of life through local recurrence, additional local therapies beyond surgical resection and ionizing radiation are also worth pursuing. The use of tumor-treating fields (TTF) that is, antimitotic treatment delivered by low-energy electric fields, is being studied in brain metastases after their demonstrated successes in glioblastoma (88, 89). A pilot study of the electromagnetic device NovoTTF-100A is underway for patients diagnosed with recurrent WHO grade 2 or grade 3 intracranial supratentorial meningioma (NCT01892397) (90). Another phase II study that combines NovoTTF-200A, which was approved by the US Food and Drug Administration for glioblastoma multiforme, with bevacizumab is currently underway for recurrent or progressive meningioma (NCT02847559) (**Table 2**). These and other studies may open the way for new modes of treatment and alternative routes of attack to target recurrent meningiomas.

## 8 Conclusion

Publication of the WHO 2021 criteria has officially moved meningioma grading into the molecular era, with recognition of the independent prognostic implications of TERT promoter mutations and CDKN2A/B deletions. Management strategies for intracranial meningiomas include observation, surgical resection, and radiotherapy, with outcomes varying depending on tumor grade. The initial management strategy depends on numerous patient- and tumor-specific factors, and owing to the diverse nature of presentation, optimal therapy should be tailored on a case-by-case basis. Better therapeutic approaches for grade 2 and 3 tumors are needed because of persistent poor outcomes. Systemic therapy has produced disappointing results for recurrent or treatment-refractory disease, however, with increasing knowledge of molecular drivers in some tumors and an increased repertoire of agents that target these drivers, there is hope for improving outcomes for patients with recurrent and aggressive meningiomas.

## Author contributions

Planning and Outline: VY. Writing, Original Draft: VY, AM, TH, and TB. Review and Editing: VY, AM, TH, TB, SM, and SR. Project Supervision: TB. All authors contributed to the article and approved the submitted version.

## References

[1] Ostrom QT Gittleman H Liao P Vecchione-Koval T Wolinsky Y Kruchko C. CBTRUS Statistical Report: Primary brain and other central nervous system tumors diagnosed in the United States in 2010-2014. Neuro Oncol (2017) 19:v1–v88. doi: 10.1093/NEUONC/NOX158 29117289 PMC5693142

[2] Hwang KL Hwang WL Bussière MR Shih HA. The role of radiotherapy in the management of high-grade meningiomas. Chin Clin Oncol (2017) 6:6–6. doi: 10.21037/CCO.V0I0.15550 28758409

[3] Louis DN Perry A Reifenberger G von Deimling A Figarella-Branger D Cavenee WK. The 2016 world health organization classification of tumors of the central nervous system: a summary. Acta Neuropathol (2016) 131:803–20. doi: 10.1007/S00401-016-1545-1 27157931

[4] Louis DN Perry A Wesseling P Brat DJ Cree IA Figarella-Branger D. The 2021 WHO classification of tumors of the central nervous system: a summary. Neuro Oncol (2021) 23:1231–51. doi: 10.1093/NEUONC/NOAB106 PMC8328013 34185076

[5] Sahm F Schrimpf D Olar A Koelsche C Reuss D Bissel J. TERT promoter mutations and risk of recurrence in meningioma. J Natl Cancer Inst (2015) 108: djv377. doi: 10.1093/JNCI/DJV377 26668184 PMC4849806

[6] Spiegl-Kreinecker S Lötsch D Neumayer K Kastler L Gojo J Pirker C. TERT promoter mutations are associated with poor prognosis and cell immortalization in meningioma. Neuro Oncol (2018) 20:1584–93. doi: 10.1093/NEUONC/NOY104 PMC6231195 30010853

[7] Sievers P Hielscher T Schrimpf D Stichel D Reuss DE Berghoff AS. CDKN2A/B homozygous deletion is associated with early recurrence in meningiomas. Acta Neuropathol (2020) 140:409–13. doi: 10.1007/S00401-020-02188-W PMC7423850 32642869

[8] Liu N Song SY Jiang JB Wang TJ Yan CX. The prognostic role of Ki-67/MIB-1 in meningioma: A systematic review with meta-analysis. Medicine (2020) 99:e18644. doi: 10.1097/MD.0000000000018644 32118704 PMC7478528

[9] Menger R Connor DE Jr. Chan AY Jain G Nanda A. Degree of resection and Ki-67 labeling index for recurring meningiomas. Cureus (2017) 9: e1820. doi: 10.7759/CUREUS.1820 29312841 PMC5752228

[10] Preusser M Brastianos PK Mawrin C. Advances in meningioma genetics: novel therapeutic opportunities. Nat Rev Neurol (2018) 14:106–15. doi: 10.1038/nrneurol.2017.168 29302064

[11] Mei Y Bi WL Greenwald NF Agar NY Beroukhim R Dunn GP. Genomic profile of human meningioma cell lines. PloS One (2017) 12:e0178322–e0178322. doi: 10.1371/journal.pone.0178322 28552950 PMC5446134

[12] Spasic M Pelargos PE Barnette N Bhatt NS Lee SJ Ung N. Incidental meningiomas: management in the neuroimaging era. Neurosurg Clin N Am (2016) 27:229–38. doi: 10.1016/J.NEC.2015.11.012 27012387

[13] Champeaux C Wilson E Shieff C Khan AA Thorne L. WHO grade II meningioma: a retrospective study for outcome and prognostic factor assessment. J Neurooncol (2016) 129:337–45. doi: 10.1007/s11060-016-2181-2 27311726

[14] Cardis E Deltour I Vrijheid M Combalot E Moissonnier M Tardy H. Brain tumour risk in relation to mobile telephone use: results of the INTERPHONE international case–control study. Int J Epidemiol (2010) 39:675–94. doi: 10.1093/IJE/DYQ079 20483835

[15] Wiemels J Wrensch M Claus EB. Epidemiology and etiology of meningioma. J Neurooncol (2010) 99:307–14. doi: 10.1007/s11060-010-0386-3 PMC2945461 20821343

[16] Ron E Modan B Boice JD Alfandary E Stovall M Chetrit A. Tumors of the brain and nervous system after radiotherapy in childhood. New Engl J Med (1988) 319:1033–9. doi: 10.1056/NEJM198810203191601 3173432

[17] Blowers L Preston-Martin S Mack WJ. Dietary and other lifestyle factors of women with brain gliomas in Los Angeles country (California, USA). Cancer Causes Control (1997) 8:5–12. doi: 10.1023/A:1018437031987 9051317

[18] Soffer D Pittaluga S Feiner M Beller AJ. Intracranial meningiomas following low-dose irradiation to the head. J Neurosurg (1983) 59:1048–53. doi: 10.3171/JNS.1983.59.6.1048 6631499

[19] Rubinstein AB Shalit MN Cohen ML Zandbank U Reichenthal E. Radiation-induced cerebral meningioma: a recognizable entity. J Neurosurg (1984) 61:966–71. doi: 10.3171/JNS.1984.61.5.0966 6593438

[20] Sadetzki S Flint-Richter P Starinsky S Novikov I Lerman Y Goldman B. Genotyping of patients with sporadic and radiation-associated meningiomas. Cancer Epidemiol Biomarkers Prev (2005) 14:969–76. doi: 10.1158/1055-9965.EPI-04-0366 15824172

[21] Agnihotri S Suppiah S Tonge PD Jalali S Danesh A Bruce JP. Therapeutic radiation for childhood cancer drives structural aberrations of NF2 in meningiomas. Nat Commun (2017) 8:1–7. doi: 10.1038/s41467-017-00174-7 28775249 PMC5543118

[22] Joachim T Ram Z Rappaport ZH Simon M Schramm J Wiestler OD. Comparative analysis of the NF2, TP53, PTEN, KRAS, NRAS and HRAS genes in sporadic and radiation-induced human meningiomas. Int J Cancer (2001) 94:218–21. doi: 10.1002/IJC.1467 11668501

[23] Shoshan Y Chernova O Jeun SS Somerville RP Israel Z Barnett GH. Radiation-induced meningioma: A distinct molecular genetic pattern? J Neuropathol Exp Neurol (2000) 59:614–20. doi: 10.1093/JNEN/59.7.614 10901233

[24] Pemov A Dewan R Hansen NF Chandrasekharappa SC Ray-Chaudhury A Jones K. Comparative clinical and genomic analysis of neurofibromatosis type 2-associated cranial and spinal meningiomas. Sci Rep (2020) 10:1–10. doi: 10.1038/s41598-020-69074-z 32724039 PMC7387487

[25] Supartoto A Mahayana IT Heriyanto DS Sasongko MB Respatika HD Sakti DH. Neurofibromatosis type 2 gene mutation and progesterone receptor messenger RNA expression in the pathogenesis of sporadic orbitocranial meningioma. Int J Ophthalmol (2019) 12:571. doi: 10.18240/IJO.2019.04.07 31024808 PMC6469556

[26] Kerr K Qualmann K Esquenazi Y Hagan J Kim DH. Familial syndromes involving meningiomas provide mechanistic insight into sporadic disease. Neurosurgery (2018) 83:1107–18. doi: 10.1093/neuros/nyy121 PMC6235681 29660026

[27] Galani V Lampri E Varouktsi A Alexiou G Mitselou A Kyritsis AP. Genetic and epigenetic alterations in meningiomas. Clin Neurol Neurosurg (2017) 158:119–25. doi: 10.1016/j.clineuro.2017.05.002 28527972

[28] Elefante A Russo C di Stasi M Vola E Ugga L Tortora F. Neuroimaging in meningiomas: old tips and new tricks. Mini-invasive Surg (2021) 5:7. doi: 10.20517/2574-1225.2020.102

[29] Afshar-Oromieh A Giesel FL Linhart HG Haberkorn U Haufe S Combs SE. Detection of cranial meningiomas: Comparison of 68Ga-DOTATOC PET/CT and contrast-enhanced MRI. Eur J Nucl Med Mol Imaging (2012) 39:1409–15. doi: 10.1007/S00259-012-2155-3/FIGURES/5 22669255

[30] Perlow HK Siedow M Gokun Y McElroy J Matsui J Zoller W. 68 ga-DOTATATE PET-based radiation contouring creates more precise radiation volumes for patients with meningioma. Int J Radiat Oncol Biol Phys (2022) 113:859–65. doi: 10.1016/J.IJROBP.2022.04.009 35460804

[31] Galldiks N Albert NL Sommerauer M Grosu AL Ganswindt U Law I. PET imaging in patients with meningioma—report of the RANO/PET Group. Neuro Oncol (2017) 19:1576. doi: 10.1093/NEUONC/NOX112 28605532 PMC5716194

[32] Zeidman LA Ankenbrandt WJ Du H Paleologos N Vick NA. Growth rate of non-operated meningiomas. J Neurol (2008) 255:891–5. doi: 10.1007/S00415-008-0801-2 18350353

[33] Apra C Peyre M Kalamarides M. Current treatment options for meningioma. Expert Rev Neurother (2018) 18:241–9. doi: 10.1080/14737175.2018.1429920 29338455

[34] Simpson D. The recurrence of intracranial meningiomas after surgical treatment. J Neurol Neurosurg Psychiatry (1957) 20:22–39. doi: 10.1136/jnnp.20.1.22 13406590 PMC497230

[35] Winther TL Torp SH. Significance of the extent of resection in modern neurosurgical practice of World Health Organization grade I meningiomas. World Neurosurg (2017) 99:104–10. doi: 10.1016/J.WNEU.2016.11.034 27867123

[36] Przybylowski CJ Hendricks BK Frisoli FA Zhao X Cavallo C Moreira LB. Prognostic value of the Simpson grading scale in modern meningioma surgery: Barrow Neurological Institute experience. J Neurosurg (2020) 135:515–23. doi: 10.3171/2020.6.JNS20374 33096534

[37] Chotai S Schwartz TH. The Simpson grading: is it still valid? Cancers (Basel) (2022) 14:2007. doi: 10.3390/CANCERS14082007 35454912 PMC9031418

[38] Brokinkel B Spille DC Brokinkel C Hess K Paulus W Bormann E. The Simpson grading: defining the optimal threshold for gross total resection in meningioma surgery. Neurosurg Rev (2021) 44:1713–20. doi: 10.1007/S10143-020-01369-1 PMC8397672 32809081

[39] Nanda A Bir SC Maiti TK Konar SK Missios S Guthikonda B. Relevance of Simpson grading system and recurrence-free survival after surgery for World Health Organization Grade I meningioma. J Neurosurg (2017) 126:201–11. doi: 10.3171/2016.1.JNS151842 27058201

[40] Sughrue ME Kane AJ Shangari G Rutkowski MJ McDermott MW Berger MS. The relevance of Simpson Grade I and II resection in modern neurosurgical treatment of World Health Organization Grade I meningiomas. J Neurosurg (2010) 113:1029–35. doi: 10.3171/2010.3.JNS091971 20380529

[41] Goldbrunner R Stavrinou P Jenkinson MD Sahm F Mawrin C Weber DC. EANO guideline on the diagnosis and management of meningiomas. Neuro Oncol (2021) 23:1821–34. doi: 10.1093/neuonc/noab150 PMC8563316 34181733

[42] Aghi MK Carter BS Cosgrove GR Ojemann RG Amin-Hanjani S Martuza RL. Long-term recurrence rates of atypical meningiomas after gross total resection with or without postoperative adjuvant radiation. Neurosurgery (2009) 64:56–60. doi: 10.1227/01.NEU.0000330399.55586.63 19145156

[43] Komotar RJ Bryan Lorgulescu J Raper DMS Holland EC Beal K Bilsky MH. The role of radiotherapy following gross-total resection of atypical meningiomas: Clinical article. J Neurosurg (2012) 117:679–86. doi: 10.3171/2012.7.JNS112113 22920955

[44] Chen WC Magill ST Wu A Vasudevan HN Morin O Aghi MK. Histopathological features predictive of local control of atypical meningioma after surgery and adjuvant radiotherapy. J Neurosurg (2018) 130:443–50. doi: 10.3171/2017.9.JNS171609 29624151

[45] Lee KD DePowell JJ Air EL Dwivedi AK Kendler A McPherson CM. Atypical meningiomas: is postoperative radiotherapy indicated? Neurosurg Focus (2013) 35. doi: 10.3171/2013.9.FOCUS13325 24289123

[46] Fioravanzo A Caffo M di Bonaventura R Gardiman MP Ghimenton C Ius T. A risk score based on 5 clinico-pathological variables predicts recurrence of atypical meningiomas. J Neuropathol Exp Neurol (2020) 79:500–7. doi: 10.1093/JNEN/NLAA018 32232472

[47] Chun SW Kim KM Kim MS Kang H Dho YS Seo Y. Adjuvant radiotherapy versus observation following gross total resection for atypical meningioma: a systematic review and meta-analysis. Radiat Oncol (2021) 16. doi: 10.1186/S13014-021-01759-9 PMC7890913 33596974

[48] Kim MS Chun SW Dho YS Seo Y Lee JH Won JK. Histopathological predictors of progression-free survival in atypical meningioma: a single-center retrospective cohort and meta-analysis. Brain Tumor Pathol (2022) 39:99–110. doi: 10.1007/S10014-021-00419-W 35031884

[49] Wang C Kaprealian TB Suh JH Kubicky CD Ciporen JN Chen Y. Overall survival benefit associated with adjuvant radiotherapy in WHO grade II meningioma. Neuro Oncol (2017) 19:1263–70. doi: 10.1093/NEUONC/NOX007 PMC5570224 28371851

[50] Kessler RA Garzon-Muvdi T Yang W Weingart J Olivi A Huang J. Metastatic atypical and anaplastic meningioma: A case series and review of the literature. World Neurosurg (2017) 101:47–56. doi: 10.1016/J.WNEU.2017.01.070 28143726

[51] Adeberg S Hartmann C Welzel T Rieken S Habermehl D von Deimling A. Long-term outcome after radiotherapy in patients with atypical and Malignant meningiomas–clinical results in 85 patients treated in a single institution leading to optimized guidelines for early radiation therapy. Int J Radiat Oncol Biol Phys (2012) 83:859–64. doi: 10.1016/J.IJROBP.2011.08.010 22137023

[52] Rogers L Zhang P Vogelbaum MA Perry A Ashby L Modi J. Low-risk meningioma: initial outcomes from NRG Oncology/RTOG 0539. Int J Radiat Oncol Biol Phys (2016) 96:939–40. doi: 10.1016/J.IJROBP.2016.09.051

[53] Rogers L Zhang P Vogelbaum MA Perry A Ashby LS Modi JM. Intermediate-risk meningioma: initial outcomes from NRG Oncology RTOG 0539. J Neurosurg (2018) 129:35–47. doi: 10.3171/2016.11.JNS161170 28984517 PMC5889346

[54] Rogers CL Won M Vogelbaum MA Perry A Ashby LS Modi JM. High-risk meningioma: initial outcomes from NRG Oncology/RTOG 0539. Int J Radiat Oncol Biol Phys (2020) 106:790–9. doi: 10.1016/J.IJROBP.2019.11.028 PMC7117785 31786276

[55] Manabe Y Murai T Ogino H Tamura T Iwabuchi M Mori Y. CyberKnife stereotactic radiosurgery and hypofractionated stereotactic radiotherapy as first-line treatments for imaging-diagnosed intracranial meningiomas. Neurol Med Chir (Tokyo) (2017) 57:627–33. doi: 10.2176/nmc.oa.2017-0115 PMC5735225 29021413

[56] Di Franco R Borzillo V Ravo V Falivene S ROmano FJ Muto M. Radiosurgery and stereotactic radiotherapy with cyberknife system for meningioma treatment. Neuroradiol J (2018) 31:18–26. doi: 10.1177/1971400917744885 29206077 PMC5790000

[57] Pollock BE Stafford SL Link MJ Garces YI Foote RL. Single-fraction radiosurgery for presumed intracranial meningiomas: efficacy and complications from a 22-year experience. Int J Radiat Oncol Biol Phys (2012) 83:1414–8. doi: 10.1016/J.IJROBP.2011.10.033 22209154

[58] Pollock BE Stafford SL Utter A Giannini C Schreiner SA. Stereotactic radiosurgery provides equivalent tumor control to Simpson Grade 1 resection for patients with small- to medium-size meningiomas. Int J Radiat Oncol Biol Phys (2003) 55:1000–5. doi: 10.1016/S0360-3016(02)04356-0 12605979

[59] Wang WH Lee CC Yang HC Liu K d Wu HM Shiau CY. Gamma knife radiosurgery for atypical and anaplastic meningiomas. World Neurosurg (2016) 87:557–64. doi: 10.1016/J.WNEU.2015.10.021 26485417

[60] Zhang M Ho AL D’Astous M Pendharkar A v. Choi CYH Thompson PA. CyberKnife stereotactic radiosurgery for atypical and Malignant meningiomas. World Neurosurg (2016) 91:574–581.e1. doi: 10.1016/J.WNEU.2016.04.019 27108030

[61] Helis CA Hughes RT Cramer CK Tatter SB Laxton AW Bourland JD. Stereotactic radiosurgery for atypical and anaplastic meningiomas. World Neurosurg (2020) 144:e53–61. doi: 10.1016/J.WNEU.2020.07.211 32758657

[62] Ding D Starke RM Hantzmon J Yen CP Williams BJ Sheehan JP. The role of radiosurgery in the management of WHO Grade II and III intracranial meningiomas. Neurosurg Focus (2013) 35. doi: 10.3171/2013.9.FOCUS13364 24289124

[63] Marchetti M Bianchi S Pinzi V Tramacere I Fumagalli ML Milanesi IM. Multisession radiosurgery for sellar and parasellar benign meningiomas: long-term tumor growth control and visual outcome. Neurosurgery (2016) 78:638–46. doi: 10.1227/NEU.0000000000001073 26492428

[64] Huang SH Wang CC Wei KC Chang CN Chuang CC Chen HC. Treatment of intracranial meningioma with single-session and fractionated radiosurgery: a propensity score matching study. Sci Rep (2020) 10. doi: 10.1038/S41598-020-75559-8 PMC7595213 33116194

[65] Nguyen EK Nguyen TK Boldt G Louie A v. Bauman GS. Hypofractionated stereotactic radiotherapy for intracranial meningioma: a systematic review. Neurooncol Pract (2019) 6:346–53. doi: 10.1093/NOP/NPY053 PMC6753355 31555449

[66] Patil CG Hoang S Borchers DJ Sakamoto G Soltys SG Gibbs IC. Predictors of peritumoral edema after stereotactic radiosurgery of supratentorial meningiomas. Neurosurgery (2008) 63:435–40. doi: 10.1227/01.NEU.0000325257.58684.92 18812954

[67] Milker-Zabel S Zabel-Du Bois A Huber P Schlegel W Debus J. Fractionated stereotactic radiation therapy in the management of benign cavernous sinus meningiomas : long-term experience and review of the literature. Strahlenther Onkol (2006) 182:635–40. doi: 10.1007/S00066-006-1548-2 17072520

[68] Morgan TM Zaenger D Switchenko JM Eaton BR Crocker IR Ali AN. Fractionated radiotherapy is associated with lower rates of treatment-related edema than stereotactic radiosurgery in magnetic resonance imaging-defined meningiomas. World Neurosurg (2019) 121:e640–6. doi: 10.1016/J.WNEU.2018.09.179 PMC6407415 30292026

[69] Hug EB DeVries A Thornton AF Munzenrider JE Pardo FS Hedley-Whyte ET. Management of atypical and Malignant meningiomas: role of high-dose, 3D-conformal radiation therapy. J Neurooncol (2000) 48:151–60. doi: 10.1023/A:1006434124794 11083080

[70] Boskos C Feuvret L Noel G Habrand JL Pommier P Alapetite C. Combined proton and photon conformal radiotherapy for intracranial atypical and Malignant meningioma. Int J Radiat Oncol Biol Phys (2009) 75:399–406. doi: 10.1016/J.IJROBP.2008.10.053 19203844

[71] Chen WC Hara J Magill ST Wu A Aghi MK Theodosopoulos P v.. Salvage therapy outcomes for atypical meningioma. J Neurooncol (2018) 138:425–33. doi: 10.1007/S11060-018-2813-9 29480505

[72] Phan J Sio TT Nguyen TP Takiar V Gunn GB Garden AS. Reirradiation of head and neck cancers with proton therapy: outcomes and analyses. Int J Radiat Oncol Biol Phys (2016) 96:30–41. doi: 10.1016/J.IJROBP.2016.03.053 27325480

[73] Koch MJ Agarwalla PK Royce TJ Shih HA Oh K Niemierko A. Brachytherapy as an adjuvant for recurrent atypical and Malignant meningiomas. Neurosurgery (2019) 85:E910–6. doi: 10.1093/NEUROS/NYZ115 PMC7308736 31329941

[74] Brachman DG Youssef E Dardis CJ Sanai N Zabramski JM Smith KA. Resection and permanent intracranial brachytherapy using modular, biocompatible cesium-131 implants: results in 20 recurrent, previously irradiated meningiomas. J Neurosurg (2018) 131:1819–28. doi: 10.3171/2018.7.JNS18656 30579269

[75] Buerki RA Horbinski CM Kruser T Horowitz PM James CD Lukas RV. An overview of meningiomas. Future Oncol (2018) 14:2161–77. doi: 10.2217/fon-2018-0006 PMC6123887 30084265

[76] Karsy M Guan J Cohen A Colman H Jensen RL. Medical management of meningiomas: current status, failed treatments, and promising horizons. Neurosurg Clin N Am (2016) 27:249–60. doi: 10.1016/j.nec.2015.11.002 27012389

[77] Kaley TJ Wen P Schiff D Ligon K Haidar S Karimi S. Phase II trial of sunitinib for recurrent and progressive atypical and anaplastic meningioma. Neuro Oncol (2015) 17:116–21. doi: 10.1093/neuonc/nou148 PMC4483051 25100872

[78] Franke AJ Skelton WP IV Woody LE Bregy A Shah AH Vakharia K. Role of bevacizumab for treatment-refractory meningiomas: A systematic analysis and literature review. Surg Neurol Int (2018) 9:133. doi: 10.4103/sni.sni_264_17 30090665 PMC6057170

[79] Shih KC Chowdhary S Rosenblatt P Weir AB Shepard GC Williams JT. A phase II trial of bevacizumab and everolimus as treatment for patients with refractory, progressive intracranial meningioma. J Neurooncol (2016) 129:281–8. doi: 10.1007/s11060-016-2172-3 27311730

[80] Lou E Sumrall AL Turner S Peters KB Desjardins A Vredenburgh JJ. Bevacizumab therapy for adults with recurrent/progressive meningioma: a retrospective series. J Neurooncol (2012) 109:63–70. doi: 10.1007/s11060-012-0861-0 22535433 PMC3404217

[81] NCCN clinical practice guidelines in oncology: central nervous system cancers, in: National comprehensive cancer network (2023). Available at: https://www.nccn.org/professionals/physician_gls/pdf/cns.pdf (Accessed May 19, 2023).

[82] Wen PY Quant E Drappatz J Beroukhim R Norden AD. Medical therapies for meningiomas. J Neurooncol (2010) 99:365–78. doi: 10.1007/s11060-010-0349-8 20820875

[83] Kaley T Barani I Chamberlain M McDermott M Panageas K Raizer J. Historical benchmarks for medical therapy trials in surgery- and radiation-refractory meningioma: a RANO review. Neuro Oncol (2014) 16:829–40. doi: 10.1093/neuonc/not330 PMC4022224 24500419

[84] Preusser M Spiegl-Kreinecker S Lötsch D Wöhrer A Schmook M Dieckmann K. Trabectedin has promising antineoplastic activity in high-grade meningioma. Cancer (2012) 118:5038–49. doi: 10.1002/cncr.27460 22392434

[85] Balik V Sulla I Park HH Sarissky M. *In vitro* testing to a panel of potential chemotherapeutics and current concepts of chemotherapy in benign meningiomas. Surg Oncol (2015) 24:292—299. doi: 10.1016/j.suronc.2015.06.004 26099192

[86] Euskirchen P Peyre M. Management of meningioma. Presse Med (2018) 47:e245–52. doi: 10.1016/j.lpm.2018.05.016 30449639

[87] Vismodegib and FAK inhibitor GSK2256098 in treating patients with progressive meningiomas, in: US national library of medicine (2020). Available at: https://clinicaltrials.gov/ct2/show/record/NCT02523014 (Accessed April 10, 2020).

[88] Stupp R Wong ET Kanner AA Steinberg D Engelhard H Heidecke V. NovoTTF-100A versus physician’s choice chemotherapy in recurrent glioblastoma: a randomised phase III trial of a novel treatment modality. Eur J Cancer (2012) 48:2192–202. doi: 10.1016/J.EJCA.2012.04.011 22608262

[89] Stupp R Taillibert S Kanner A Read W Steinberg DM Lhermitte B. Effect of tumor-treating fields plus maintenance temozolomide vs maintenance temozolomide alone on survival in patients with glioblastoma: A randomized clinical trial. JAMA (2017) 318:2306–16. doi: 10.1001/JAMA.2017.18718 PMC5820703 29260225

[90] Kaley T. Pilot study of optune (NovoTTF-100A) for recurrent atypical and anaplastic meningioma. Available at: https://clinicaltrials.gov/ct2/show/study/NCT01892397 (Accessed November 29, 2022).

